# The individual and combined associations of health behaviours with health-related quality of life amongst junior high school students in China

**DOI:** 10.3389/fpubh.2023.1283721

**Published:** 2023-10-12

**Authors:** Ze Hua Liu, Yi Lin Wang, Yue Shuang Yu, Yan Ren, Tong Zhang, Hong Qing Liu, Xiu Yun Wu

**Affiliations:** ^1^School of Public Health, Weifang Medical University, Weifang, Shandong, China; ^2^Affiliated Hospital of Jining Medical University, Jining, Shandong, China

**Keywords:** sedentary behaviour, sleep, physical activity, breakfast, health-related quality of life, junior high school students

## Abstract

**Objectives:**

This study aimed to investigate the individual and joint associations of sedentary behaviour, physical activity (PA), sleep and breakfast eating on health-related quality of life (HRQoL) amongst Chinese junior high school students.

**Methods:**

Data were from 783 junior high school students who participated in a health behaviour and health survey in Jining city of Shandong province of China. HRQoL was measured by the EuroQol five-dimensional questionnaire, youth version (EQ-5D-Y). Multivariable logistic and linear regressions were applied to examine the associations between health behaviours and HRQoL.

**Results:**

Multivariable regression analyses showed that using a computer ≥ 2 h a day (vs. < 2 h/day) is associated with increased likelihood of having health problems in the three EQ-D-Y dimensions, including walking, looking after self and doing usual activities. Lower PA is associated with more problems in feeling worried, sad or unhappy, and with a lower visual analogue scale (VAS) score. Students who had insufficient sleep time (e.g., <7 h/day), and skipped eating breakfast were more likely to experience lower HRQoL in the dimensions of having pain or discomfort, and feeling worried, sad or unhappy, and a lower VAS score than those students who had longer sleep time and no breakfast skipping. Students who reported having the combined ≥ 2 unhealthy behaviours relative to the peers with 0–1 unhealthy behaviours were more likely to have lower HRQoL.

**Conclusion:**

The findings in the present study underline the importance of promoting healthy behaviours in order to improve HRQoL amongst Chinese junior high school students.

## 1. Introduction

Health-related quality of life (HRQoL) is a subjective evaluation of an individual’s overall health status and its underlying sub-dimensions, including physical, psychological and social functioning and well-being (1). Evaluations of HRQoL amongst children and adolescents is critical in order to develop effective and targeted interventions to enhance their health status. Previous studies have shown that health-related behaviours such as sedentary behaviours, physical activity (PA), sleep and diet are associated with HRQoL amongst children and adolescents (2–4). Research evidence suggests that high physical activity is associated with better HRQoL, and sedentary behaviour is related to lower HRQoL amongst children and adolescents (2). A lack of sleep and skipping breakfast are related to lower HRQoL amongst children and youth (3–6). However, the relationships between the health behaviours and HRQoL have been mostly investigated in developed countries (2, 3). Very few studies have examined the influence of PA, sedentary behaviour, breakfast skipping and/or sleep on HRQoL in adolescents in China (7). Besides, some studies documented that health behaviours may not act in isolation in their associations with health outcomes, and that the co-occurrence of multiple health behaviours may cause greater effects than their individual effect (8, 9). Few studies have yet investigated the influence of combined or joint associations of PA, sedentary behaviours, sleep duration and diet behaviours with HRQoL in school-aged children and youth (8, 10). To our knowledge, no study has been found to examine the combined effect of multiple health behaviours, including PA, sedentary behaviours, sleep and breakfast eating habit simultaneously on HRQoL in China. Understanding the combined impact of multiple health behaviours on HRQoL will help address synergistic effects between the health behaviours and health outcomes, and thus provide scientific information for public health policy makers in prioritising school-based health intervention programmes in order to promote healthy lifestyle behaviours and health amongst adolescents. Given prior studies have mainly examined the impact of single health behaviour on health, further interventions of the joint association between multi-dimensional health behaviours and HRQoL will help address cumulative benefits of health behaviours for adolescents’ health and wellbeing.

This study aimed to investigate associations between physical activity, sedentary behaviour, sleep time, breakfast eating and HRQoL; and to examine combined associations of the health-related behaviours on HRQoL amongst junior high school students in Jining city, Shandong province of China.

## 2. Materials and methods

### 2.1. Participants

This study surveyed students from four junior high schools in Jining city, Shandong province of China in June and July in 2020. Jining city is one of the historical and large cities located in the southwest region in the province with a population in urban area of approximately five millions in 2022.^1^ The survey aimed to evaluate health-related behaviours and health amongst school students to promote their healthy lifestyle behaviours and health status. Four junior high schools were selected in the city. Classes were then randomly selected within each of the chosen schools. In total, 800 students aged 11–16 years from 16 classes in the four schools were invited to participate, and 783 students (98%) completed the survey with complete information on lifestyle behaviours and HRQoL. The survey included the EuroQol five-dimensional questionnaire, youth version (EQ-5D-Y) with a five-dimensional descriptive system and a visual analogue scale (VAS) (11), questions on sedentary behaviours (using a computer, using a cellphone), physical activity, sleep and dietary behaviours (e.g., frequency of eating breakfast), and socio-demographic characteristics of students.

### 2.2. Measures of health-related quality of life

HRQoL was measured by the EQ-5D-Y designed for use amongst children and youth aged between 8 and 18 years. The EQ-5D-Y instrument has questions on five-dimensions: walking; looking after myself; doing usual activities; having pain or discomfort; and feeling worried, sad or unhappy (11). Each dimension has three response options asking whether children have ‘no problems, some problems or a lot of problems’. The instrument also includes VAS which is anchored at 100 (best imaginable health) and 0 (worst imaginable health) to capture self-rated values of overall health status. The EQ-5D-Y has been validated in a number of languages and countries (12, 13). The Chinese version of the EQ-5D-Y used in this study has also been validated and showed feasible and valid for assessing HRQoL amongst children and adolescents in China (14, 15). In this study, the reliability coefficient (Cronbach’s alpha) is 0.70.

### 2.3. Assessments of lifestyle behaviours

Students were asked to report frequencies of their engagements in moderate and vigorous physical activity (MVPA) outside of schools in the past 7 days with the response levels: never or once a month, Once a week, 2–3 times a week and ≥4 times a week. In the analysis, the last two levels were combined into one group: ≥2 times a week. The sedentary behaviours were reported as daily hours spent on using a computer and playing a cellphone with response categories: less than 1 h a day, 1–2 h a day, and ≥2 h a day. Sedentary behaviours were dichotomized as <2 h/day and ≥2 h/day according to the physical activity guidelines for children and adolescents in China (16). Questions on physical activity have been previously validated, and showed a good validity and reliability amongst Chinese children and adolescents (17).

Sleep time was reported as habitual number of hours a day spent on sleep, and it was categorised to two levels: ≥7 h/day and <7 h/day. Eating behaviour was measured as frequencies of eating breakfast with 3 options: eating breakfast every day, eating breakfast often, and never or almost never eating breakfast.

### 2.4. Socio-demographic characteristics

Students’ socio-demographic characteristics included gender, grade, highest level of parental education. Grade was categorised as junior (grade 1 and 2 in junior high school) and senior (grade 3 and 4 in junior high school) levels. Parental educational attainment was categorised as junior high school or less; high school; and university/college or higher.

### 2.5. Statistical analyses

Descriptive analyses were used to describe the frequency distribution for categorical variables and the mean score with its standard deviation (SD) for VAS score. We used a chi-square test to examine differences in the prevalence of health problems for each of the five EQ-5D-Y dimensions by the health behaviour and socio-demographic variables, respectively. As very few students reported ‘a lot of problems’ in the EQ-5D-Y dimensions, we combined this with the response level ‘some problems’ into one group. A dichotomous outcome (Having any problems vs. no problems) was used in the analysis for the five EQ-5D-Y dimensions. We applied t-test (for two groups) or analysis of variance (ANOVA; for more than two groups) to examine differences in the mean VAS score by the health behaviour and socio-demographic variables.

The present study used univariate and multivariable logistic regressions, respectively, to examine the associations between the health behaviours and HRQoL measured by each of the five EQ-5D-Y dimensions. Univariate and multivariable linear regressions were applied to assess the association of the health behaviours with the VAS score. The multivariable regression analyses adjusted the confounding influence of gender, grade level and parental education.

To analyse the combined associations of health behaviours with HRQoL in regression analyses, the three-category MVPA and breakfast eating variables were dichotomized, respectively. MVPA was dichotomized as high PA (≥2 times a week) and low PA (combined ‘never or once a month’ and ‘once a week’). Breakfast eating was dichotomized as eating every day and often (healthy eating) and never or almost never eating (unhealthy eating). Using all the five dichotomous health behaviour variables (recoded as 0 = healthy, 1 = unhealthy), the total number of variables that met the unhealthy or poor level of the behaviours (from zero, one up to five) was calculated for each student observation. Next, a new health behaviour variable representing the number of combined unhealthy behaviours was generated, and categorised into three levels: 0–1, 2 and ≥3 unhealthy behaviours. The observations with zero and one unhealthy behaviour were combined since the number of students who responded to zero level was low (16.6%). Subsequently, using the combined HB variable as an exposure and HRQoL as an outcome, univariate and multivariable regressions were fitted to examine the combined effect of the health behaviours on the HRQoL outcomes.

### 2.6. Ethical approval

The present study, including the data collection was approved the Human Research Ethics Boards of the Weifang Medical University. Written informed consent to participate in this study was provided by the participants’ legal guardian/next of kin, and students themselves. All methods were performed in accordance with the relevant guidelines and regulations.

## 3. Results

Table 1 shows the frequency distribution of socio-demographic characteristics, and health behaviours of the junior high school students. Amongst 783 students, 54.5% were boys, 61.0% were in junior grade level. Approximately half of students (47.1%) engaged in MVPA ≥ 2 times a week and had sleep time less than 7 h a day (47.3%), respectively. Students who reported spending 2 h or more a day on using a cellphone and a computer accounted for 33.6% and 17.6%, respectively. Never or almost never eating breakfast was reported amongst 14.8% of students.

**Table 1:** Frequency distributions of socio-demographic characteristics, health behaviours of the junior high school students.

Variable	Frequency	Percentage, %
**Using a computer**		
<2 h/day	645	82.4
≥2 h/day	138	17.6
**Using a cellphone**		
<2 h/day	520	66.4
≥2 h/day	263	33.6
**MVPA**		
≥2 times a /week	369	47.1
Once a week	179	22.9
Never or once a month	235	30.0
**Eating breakfast**		
Eating everyday	441	56.3
Eating often	226	28.9
Never or almost never eating	116	14.8
**Sleep time**		
≥7 h/day	404	52.7
<7 h/day	362	47.3
**Number of health behaviours with the unhealthy level response**		
0–1	380	49.6
2	218	28.5
≥3	168	21.9
**Gender**		
Boys	427	54.5
Girls	356	45.5
**Grade level**		
Junior (grade 1 and 2)	478	61.0
Senior (grade 3 and 4)	305	39.0
**Parents’ education**		
Junior high school or less	158	20.2
High school	398	50.8
University/college or higher	227	29.0

MVPA, moderate and vigorous physical activity.

The prevalence of students who reported having ‘some or a lot of problems’ on the EQ-5D-Y dimensions was 5.9%, 5.2%, 12.4%, 26.4%, and 37.7% for ‘walking’, ‘looking after self’, ‘doing usual activities’, ‘having pain or discomfort’, and ‘feeling worried, sad or unhappy’. The mean VAS score was 82.0 (standard deviation, SD:18.6).

**Figure 1:**
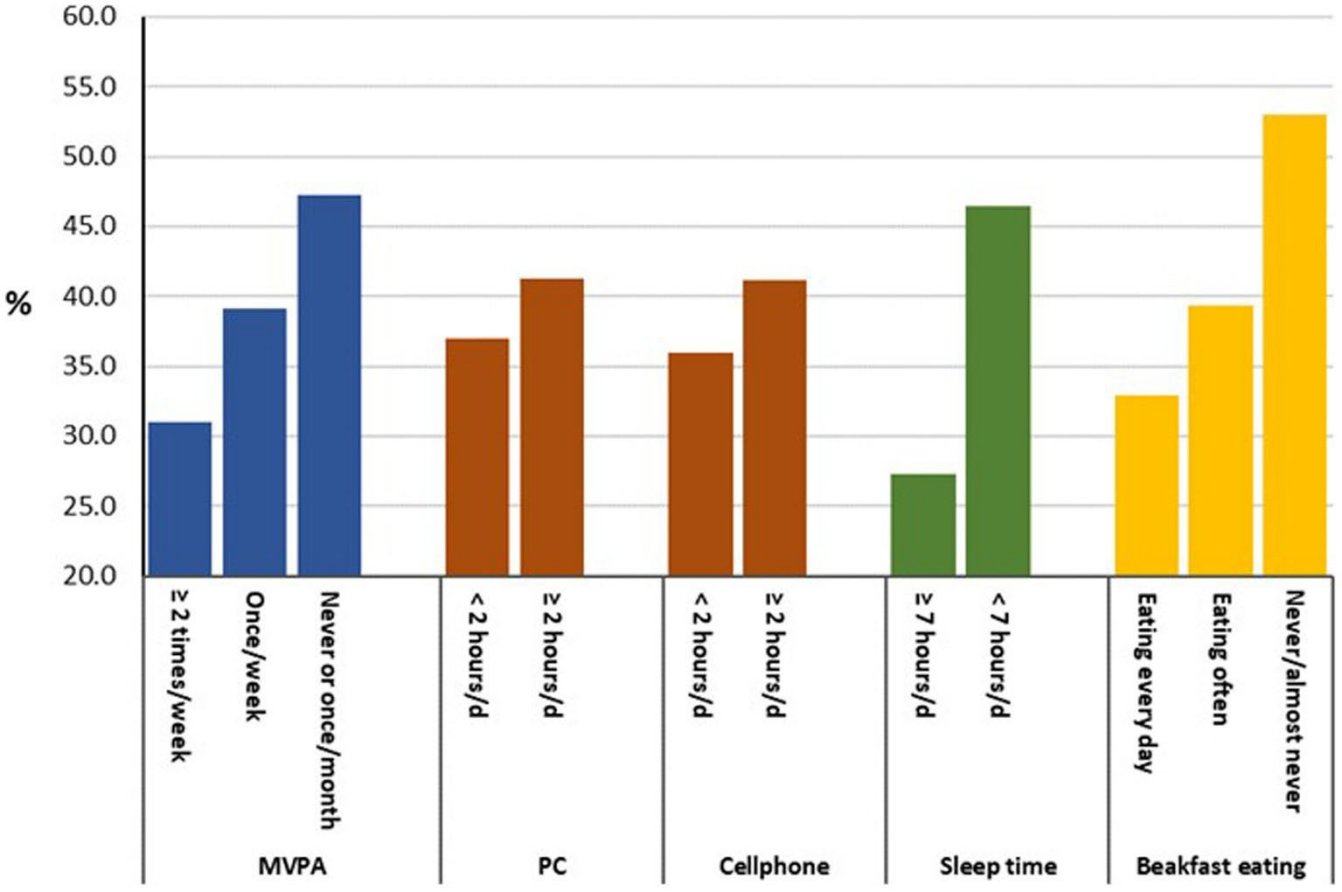
Prevalence of having some and a lot of problems in the EQ-5D-Y dimension of feeling worried, sad or unhappy by the health behaviours. MVPA, moderate and vigorous physical activity; PC, using a computer; d, day.

Table 2 presents the prevalence of problems in the EQ-5D-Y dimensions and the mean VAS score by the health behaviour and socio-demographic characteristics. Students who spent more time on using a computer or a cellphone (≥2 h/day) had a higher prevalence of health problems on ‘walking’, ‘looking after self’, and ‘doing usual activities’ in the EQ-5D-Y than students who spent less time on the sedentary behaviours (<2 h/day). In addition, using a cellphone is significantly associated with a higher prevalence of poor health problems on ‘having pain or discomfort’. Students who engaged in lower level of physical activity (MVPA < 2 times a week) had more poor health problems in three EQ-5D-Y dimensions: looking after self, having pain or discomfort and feeling worried, sad or unhappy than students who were physically active. Students who had shorter sleep time (<7 h/day) or a breakfast skipping reported more health problems in the EQ-5D-Y dimensions of ‘having pain or discomfort’ and ‘feeling worried, sad or unhappy’ than students who had longer sleep time (≥7 h/day) and ate breakfast every day. The prevalence of having some or a lot of problems in the ‘feeling worried, sad or unhappy’ dimension by the health behaviours is displayed in Figure 1.

**Table 2:** Prevalence of problems in the EQ-5D-Y dimensions and mean VAS score by health behaviours and socio-demographic characteristics amongst junior high school students.

Variable	The EQ-5D-Y dimensions (percentage of students reporting some or a lot of problems)					VAS score
	Walking	Looking after self	Doing usual activities	Having pain or discomfort	Feeling worried, sad or unhappy	Mean (95% CI)
**Using a computer**	***P* < 0.001**	***P* < 0.001**	***P* < 0.001**	*P* = 0.337	*P* = 0.339	*P* = 0.250
<2 h/day	4.0	3.7	10.2	25.7	37.0	82.4 (81.0, 83.9)
≥2 h/day	14.5	12.3	22.5	29.7	41.3	80.4 (77.2, 83.7)
**Using a cellphone**	***P* < 0.001**	***P* < 0.001**	***P* = 0.002**	***P* = 0.005**	*P* = 0.170	***P* = 0.042**
<2 h/day	3.7	3.1	9.8	23.3	36.0	83.0 (81.5, 84.6)
≥2 h/day	10.3	9.5	17.5	32.7	41.1	80.2 (77.8, 82.6)
**MVPA**	*P* = 0.058	***P* = 0.025**	*P* = 0.065	***P* = 0.016**	***P* < 0.001**	***P* < 0.001**
≥2 times a week	3.8	3.0	9.5	22.8	31.0	85.2 (83.6, 86.9)
Once a week	8.4	7.8	14.5	25.1	39.1	80.8 (78.1, 83.4)
Never or once a month	7.2	6.1	15.3	33.2	47.2	78.1 (75.4, 80.8)
**Sleep time**	*P* = 0.078	*P* = 0.740	*P* = 0.321	***P* < 0.001**	***P* < 0.001**	***P* < 0.001**
≥7 h/day	3.0	3.4	9.4	18.3	27.3	86.6 (85.2, 88.0)
<7 h/day	5.5	3.0	11.6	32.0	46.4	78.2 (76.0, 80.3)
**Eating breakfast**	*P* = 0.815	*P* = 0.792	*P* = 0.980	***P* = 0.001**	***P* < 0.001**	***P* < 0.001**
Eating every day	5.4	5.7	12.2	22.2	32.9	84.7 (83.1, 86.3)
Eating often	6.2	4.4	12.4	27.9	39.4	80.8 (78.4, 83.2)
Never or almost never eating	6.9	5.2	12.9	39.7	53.0	74.4 (70.5, 78.4)
**Number of combined unhealthy level of behaviours**	***P* < 0.001**	***P* = 0.017**	***P* = 0.002**	***P* < 0.001**	***P* < 0.001**	***P* < 0.001**
0–1	1.3	1.8	6.6	18.2	28.2	86.9 (85.4, 88.3)
2	5.1	3.2	13.8	27.5	38.5	80.3 (77.6, 83.0)
≥ 3	9.5	6.6	14.9	36.3	51.8	76.0 (72.8, 79.2)
**Gender**	*P* = 0.134	*P* = 0.069	*P* = 0.372	*P* = 0.076	***P* = 0.001**	***P* = 0.029**
Girls	4.5	3.7	11.2	29.5	44.2	80.5 (78.4, 82.6)
Boys	7.0	6.6	13.4	23.9	32.3	83.4 (81.7, 85.1)
**Grade level**	***P* = 0.031**	***P* = 0.009**	*P* = 0.400	***P* = 0.011**	*P* = 0.656	*P* = 0.694
Junior (grade 1 and 2)	7.3	6.9	13.2	23.2	37.1	81.9 (80.1, 83.7)
Senior (grade 3 and 4)	3.6	2.6	11.2	31.5	38.7	82.4 (80.6, 84.2)
**Parents’ education**	*P* = 0.201	***P* = 0.042**	*P* = 0.076	*P* = 0.795	*P* = 0.318	*P* = 0.528
Junior high school or less	8.9	8.9	16.5	24.7	34.8	80.6 (77.2, 83.9)
High school	5.0	5.0	12.8	27.4	40.3	82.4 (80.6, 84.2)
University/College or higher	5.3	3.1	8.8	26.0	35.2	82.5 (80.4, 84.7)

A bold *p*-value indicates a statistical significance (*p* < 0.05) between groups of the variables. MVPA, moderate and vigorous physical activity.

**Figure 2:**
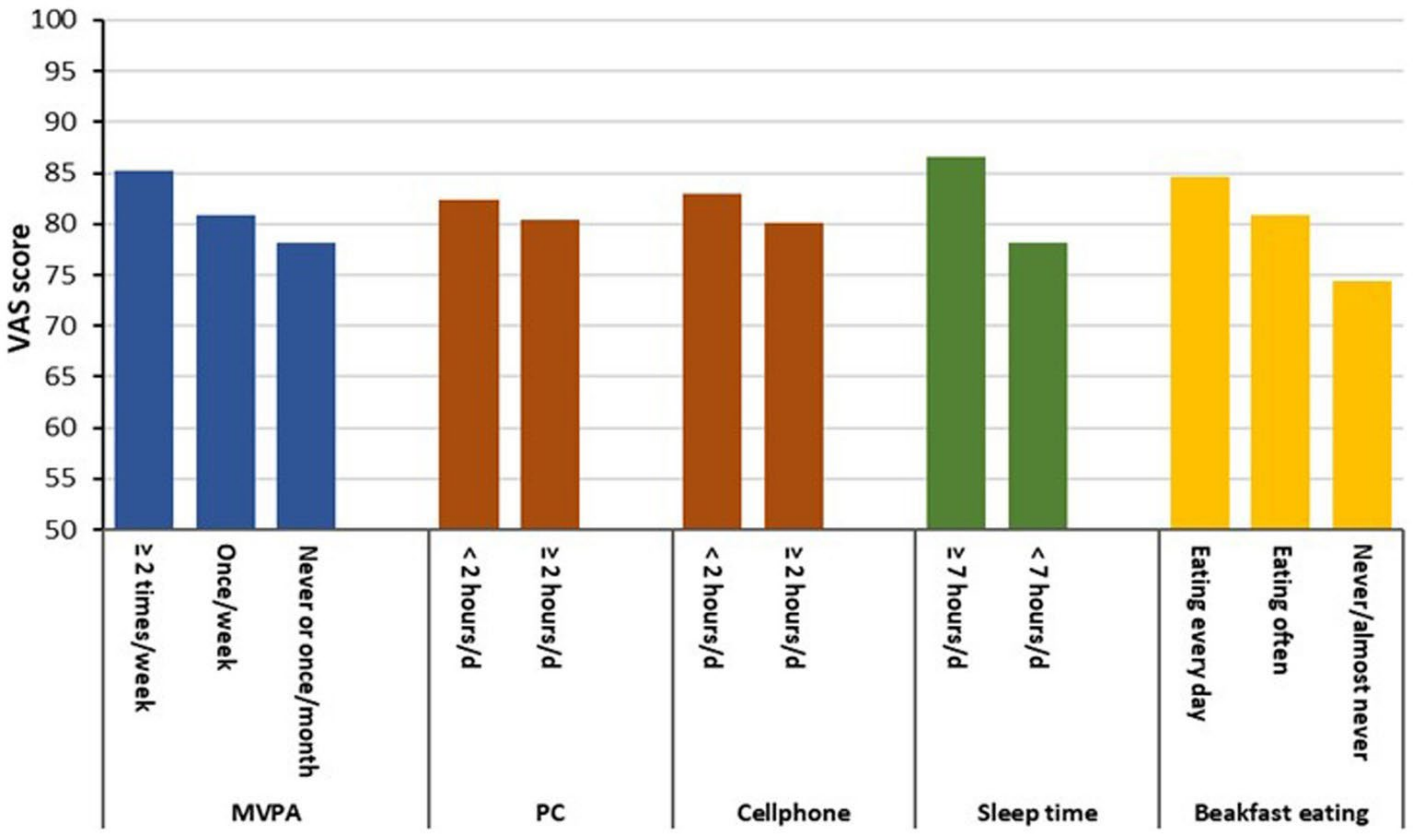
Mean VAS scores by the health behaviours. MVPA, moderate and vigorous physical activity; PC, using a computer; d, day.

Students who were inactive, used a cellphone more often, spent less time on sleep and skipped a breakfast had a lower VAS score (*p* < 0.05) relative to their reference peers. Students who had combined unhealthy behaviours on two, three and more behaviours (compared with 0-1unhealthy behaviour) had a higher prevalence of poor health problems on all five dimensions of the EQ-5D-Y, and a lower VAS score. Female students had a higher prevalence of health problems in the ‘feeling worried, sad or unhappy’, and a lower VAS score than male peers. Students in the senior grade level were affected by more health problems in ‘having pain or discomfort’ relative to students in the junior grade level. However, senior grade level students had a lower prevalence of health problems in ‘walking’ and ‘looking after self’ than junior grade level students. Higher level of parental education was associated with a lower prevalence of health problems in ‘looking after self’ amongst students. The mean VAS scores by the health behaviour variables are presented in Figure 2.

Table 3 shows the multivariable regression results for the association between health behaviours and HRQoL. Of 783 students, 17 students had missing responses to sleep time, resulting in 766 observations available in the multivariable regression analysis. After adjusting for the effect of sociodemographic and other health behaviour variables in the table, using a computer significantly affected three of the EQ-5D-Y dimensions: walking, looking after self and doing usual activities. Low MVPA was significantly associated with problems in ‘feeling worried, sad or unhappy’ (OR = 1.51, 95% CI 1.05, 2.17). Students who had sleep time < 7 h/day and skipped a breakfast were more likely to report some or a lot of problems in ‘having pain or discomfort’ and ‘feeling worried, sad or unhappy’. Regarding VAS score, low MVPA level, shorter sleep time and skipping a breakfast, respectively, correlates with a lower VAS score. Girls were more likely to feel worried, sad or unhappy than boys (OR = 1.44, 95% CI 1.04, 1.98). Students in higher grade level were more likely to experience pain or discomfort (OR = 1.65, 95% CI 1.15, 2.38).

**Table 3:** Multivariable logistic and linear regression results for the association between health behaviours and the EQ-5D-Y dimensions and VAS score amongst junior high school students.

Variable	Walking	Looking after self	Doing usual activities	Having pain or discomfort	Feeling worried, sad or unhappy	VAS score
	OR (95% CI)	OR (95% CI)	OR (95% CI)	OR (95% CI)	OR (95% CI)	Coefficient (95% CI)
**Using a computer**						
≥2 h/day	**7.31 (2.72, 19.65)**	**8.08 (2.70, 24.20)**	**3.17 (1.66, 6.08)**	1.08 (0.65, 1.79)	1.26 (0.79, 2.02)	−1.28 (−5.11, 2.56)
**Using a cellphone**						
≥2 h/day	0.57 (0.20, 1.57)	0.44 (0.14, 1.37)	0.80 (0.43, 1.48)	1.33 (0.88, 2.02)	0.92 (0.63, 1.36)	0.23 (−2.90, 3.36)
**MVPA**						
Once a week	1.61 (0.62, 4.20)	1.43 (0.48, 4.21)	1.13 (0.60, 2.12)	0.90 (0.56, 1.42)	1.15 (0.77, 1.72)	−3.02 (−6.26, 0.23)
Never or once a month	1.45 (0.60, 3.49)	1.79 (0.67, 4.75)	1.39 (0.80, 2.42)	1.30 (0.87, 1.93)	**1.51 (1.05, 2.17)**	**−4.79 (−7.79, −1.79)**
**Sleep time**						
<7 h/day	1.91 (0.86, 4.25)	0.88 (0.37, 2.14)	1.19 (0.73, 1.96)	**1.67 (1.18, 2.38)**	**1.99 (1.45, 2.74)**	**−6.74 (−9.34, −4.14)**
**Eating breakfast**						
Eating often	1.68 (0.72, 3.94)	1.25 (0.48, 3.23)	1.19 (0.69, 2.04)	**1.50 (1.01, 2.23)**	1.29 (0.91, 1.83)	**−3.63 (−6.53, −0.74)**
Never or almost never eating	1.77 (0.63, 5.03)	1.72 (0.53, 5.61)	1.17 (0.59, 2.32)	**2.16 (1.35, 3.44)**	**1.95 (1.25, 3.04)**	**−8.60 (−12.33, −4.87)**
**Gender**						
Girls vs. boys	0.48 (0.21, 1.12)	0.40 (0.15, 1.08)	0.83 (0.50, 1.38)	1.19 (0.83, 1.69)	**1.44 (1.04, 1.98)**	−0.93 (−3.55, 1.70)
**Grade level**						
Senior (grade 3 and 4)	0.49 (0.19, 1.24)	0.39 (0.12, 1.23)	1.08 (0.64, 1.83)	**1.65 (1.15, 2.38)**	1.03 (0.74, 1.43)	1.00 (−1.70, 3.70)
**Parents’ education**						
High school	0.61 (0.25, 1.53)	0.59 (0.23, 1.55)	0.82 (0.46, 1.46)	1.31 (0.82, 2.08)	1.41 (0.93, 2.12)	0.67 (−2.63, 3.97)
University/College or higher	1.10 (0.40, 3.04)	0.58 (0.17, 1.96)	0.60 (0.30, 1.20)	1.20 (0.71, 2.00)	1.21 (0.76, 1.93)	−0.57 (−4.26, 3.13)
**Constant**	NA	NA	NA	NA	NA	90.28 (86.59, 93.97)

Bold numbers indicate a statistically significance (*p* < 0.05). The reference group for the variables: MVPA: ≥ 2 times a week; using a computer/cellphone: < 2 h/day; sleep time: ≥ 7 h/day; eating breakfast: eating every day; grade level: junior (grade 1 and 2); parental education: junior high school or less. NA, not applicable.

Table 4 presents the multivariable regression results for the combined effects of multiple health behaviours (combination of MVPA, using a computer and a cellphone, sleep time and breakfast eating) and HRQoL. Compared to the reference group with relatively healthy level for the combined behaviours, students who reported the unhealthy level on three or more behaviours had a higher likelihood of experiencing lower HRQoL on all the five dimensions and the VAS score of the EQ-5D-Y. Students who responded to the poor health level on two behaviours were more likely to have lower HRQoL on all the dimensions and the VAS score except for the dimension looking after self. A dose–response relationship between the exposure level of the combined HBs and HRQoL outcomes was observed on all the EQ-5D-Y dimensions and the VAS score. For example, the VAS score of students with unhealthy behaviours of three and more was 6.34 points lower than students with two unhealthy behaviours, and 10.7 points lower than students with healthier behaviours (Table 4).

**Table 4:** Multivariable logistic and linear regression results for the association between the combined health behaviours and the EQ-5D-Y dimension and VAS score amongst junior high school students.

Variable	Walking	Looking after self	Doing usual activities	Having pain or discomfort	Feeling worried, sad or unhappy	VAS score
	OR (95% CI)	OR (95% CI)	OR (95% CI)	OR (95% CI)	OR (95% CI)	Coefficient (95% CI)
**Number of health behaviours with the unhealthy level response**						
2	**4.25 (1.45, 12.51)**	1.83 (0.63, 5.35)	**2.27 (1.29, 3.99)**	**1.73 (1.16, 2.59)**	**1.55 (1.09, 2.22)**	**−6.34 (−9.33, −3.36)**
≥3	**8.57 (3.05, 24.08)**	**3.88 (1.46, 10.35)**	**2.51 (1.39, 4.55)**	**2.60 (1.72, 3.94)**	**2.63 (1.80, 3.85)**	**−10.70 (−13.95, −7.44)**
**Gender**						
Girls vs. boys	**0.42 (0.19, 0.94)**	**0.36 (0.14, 0.93)**	0.76 (0.47, 1.23)	1.24 (0.88, 1.74)	**1.60 (1.18, 2.17)**	−2.38 (−4.94, 0.17)
**Grade level**						
Senior (grade 3 and 4)	0.43 (0.18, 1.06)	0.34 (0.11, 1.04)	0.99 (0.60, 1.64)	**1.69 (1.19, 2.39)**	1.13 (0.82, 1.55)	−0.08 (−2.60, 2.76)
**Parents’ education**						
High school	0.57 (0.23, 1.39)	0.59 (0.23, 1.51)	0.83 (0.46, 1.47)	1.25 (0.79, 1.97)	1.32 (0.88, 1.99)	1.10 (−2.25, 4.45)
University/college or higher	1.04 (0.39, 2.77)	0.57 (0.18, 1.86)	0.62 (0.31, 1.24)	1.13 (0.68, 1.88)	1.13 (0.71, 1.78)	0.15 (−3.59, 3.90)
Constant	NA	NA	NA	NA	NA	**87.23 (83.84, 90.62)**

Bold numbers indicate a statistically significance (*p* < 0.05). The reference group for the variables: 0–1 for number of health behaviours with the unhealthy level response; grade level: junior (grade 1 and 2); parental education: junior high school or less. NA, not applicable.

## 4. Discussion

The present study reveals that using a computer or a cellphone, physical activity, sleep duration and breakfast eating are associated with HRQoL in junior high school students. Students who were physically inactive, engaged in a higher level of sedentary behaviour, had shorter sleep time, and/or a higher frequency of skipping breakfast had a lower HRQoL. The associations are independent of the effect of students’ gender, grade level and parental education. This study also finds that the combined unhealthy levels of the health behaviours are associated with lower HRQoL. The dose–response relationship observed between the joint health behaviours and HRQoL suggests that a synergistic effect of multiple behaviours on HRQoL may exist amongst students.

Adolescence is an important period to establish healthy lifestyle behaviours, and it is also a period to be affected by various health problems such as poor mental health and well-being (18). Considering that adolescents’ health behaviours can persist into adulthood (19), investigations of the impact of health behaviours on HRQoL is crucial in informing public health intervention programmes amongst the young population. This study found that junior high school students who were physically active had significantly better HRQoL (VAS) scores, and a lower likelihood of feeling worried, sad or unhappy than those students who were physically inactive. This is consistent with other studies in other countries (20–23) showing that PA is related to mental health aspect of HRQoL. Similarly, the associations between increased screen time behaviours (using a computer or a cellphone) and poorer HRQoL observed in the present study are in line with other studies amongst school-age children and adolescents (20, 22, 24, 25).

To the best of our knowledge, the present study is the first to reveal the associations of sedentary behaviour, physical activity, eating breakfast and sleep time simultaneously with HRQoL amongst junior high school students in China. The study observed that students with skipping breakfast, and shorter sleep time were more likely to have poor HRQoL, including VAS score and two major domains (having pain or discomfort, and feeling worried, sad or unhappy) of the EQ-5D-Y. The finding for skipping breakfast is consistent with few other studies in Japan showing that children and adolescents who ate breakfast seldom or never had a higher odds of experiencing poor HRQoL than their peers who ate breakfast often or every day (6, 26). The association of skipping breakfast with poor HRQoL in the mental health domain, ‘feeling worried, sad or unhappy’ is in accordance with some previous studies reporting that breakfast skipping correlated with increased mental and psychological health problems (27–30). In line with previous literature (5, 7, 31–33), the results in this study showed that insufficient sleep time was related to lower HRQoL compared to longer sleep time (≥7 h/day), further highlighting the importance of better sleep duration for HRQoL in students. Notably, our observation that a lack of sleep is related to ‘feeling worried, sad or unhappy’ in the EQ-5D-Y amongst students coincides with other studies showing that poor sleep is associated with more mental health problems (24, 34, 35).

The present study contributes to the related research by revealing the correlations of a combination of certain health behaviours with HRQoL amongst junior high school students. Whilst an increasing studies have emerged over the past decade to examine the combination effect of multiple types of health behaviours such as physical activity, sedentary behaviour, sleep and/or diet on other health indicators (e.g., obesity, adiposity, cardiometabolic disorders, school academic performance, and mental health) amongst children and youth (8, 9, 34, 36), very few studies have investigated the effect of adhering to several modifiable health behaviours on HRQoL in the general populations of children and adolescents. We have found only two studies that evaluated the combined effect of these behaviours (physical activity, sedentary behaviour, sleep and diet) on HRQoL amongst adolescents (4, 31). A study in Spain found that adolescents adhering to two or more healthy levels of the behaviours (physical activity, Mediterranean diet, sleep quality, sleep duration and screen time) had higher HRQoL compared with adolescents with unhealthy levels of the behaviours (i.e., healthy lifestyle index score = 0) (31). Another study observed that Portuguese adolescents who achieved more healthy lifestyle behaviours (i.e., healthy lifestyle composite score = 6) amongst six health-related behaviours (physical activity, screen time, sleep duration, daily fruit and vegetable consumption, drinking alcohol, and smoking) had higher HRQoL relative to those who achieved less than six healthy behaviours (4). The observation in this study that adolescents who had the unhealthy level of behaviours ≥2 showed lower HRQoL compared to those with no or only one of the poor behaviours is consistent with the previous studies (4, 31). This study supports the prior research finding that the combined health-related behaviours may pose a greater impact on HRQoL than single behaviour (4, 10, 31). The present study filled the gap in the literature by investigating a cumulative effect of multiple health behaviours on HRQoL amongst junior high school students in China. It is also worth to mention that the present study analysed HRQoL outcomes, including both the overall QoL (VAS score) and HRQoL in the EQ-5D-Y dimensions, and examined the effects of both single and multifaceted health-related behaviours. Therefore, the findings in this study contributes to the literature by providing relatively a comprehensive view of the relationships between health behaviours and HRQoL amongst adolescents.

Regarding the effects of socio-demographic factors on HRQoL, the present study found a significant association between gender, grade level and HRQoL in students. The findings that girls were more likely than boys to feel worry, sad or unhappy, and senior students were more likely than junior students to show pain and discomfort problems are consistent with previous studies (37, 38) showing that girls and older students tend to have lower HRQoL relative to boys and younger students. The association between parental education and HRQoL in children and appears more inconsistent. Some studies found a significant association between parental education and HRQoL in adolescents (38), whilst other studies did not observe a significant association (31). Our finding is consistent with the later in that we did not find a significant association between parental education and HRQoL amongst students in the adjusted regression model. Future research is needed to better elucidate the relationship between parents’ educational attainment and HRQoL amongst junior high school students. Particularly, future studies that expand investigations amongst junior high school students in other regions of China and other countries with various socio-economic and cultural background will help provide better insights on the relationship of socio-economic factors with HRQoL.

The feasibility, reliability and validity of the EQ-5D-Y have been tested amongst children and youth in more than 15 countries (12). However, as the EQ-5D-Y is relatively a new HRQoL measure, very few studies have applied the EQ-5D-Y in HRQoL assessment amongst children and adolescents in China (12). The present study supports the feasibility, reliability and validity of the EQ-5D-Y amongst Chinese junior high school students. Of the 783 students, there is only one student with missing value in the dimension of ‘feeling worried, sad or unhappy’, confirming its feasibility. The Cronbach’s alpha is 0.70 in this study, showing a good reliability. The pattern of prevalence of problems in the five dimensions (e.g., higher prevalence of problems in the last two dimensions and lower prevalence of problems in the first, second and third dimensions) is similar to other studies in China and other countries like European countries (i.e., Germany, Spain, Sweden), Canada, South Africa, etc. (12–15, 39), and within the expectation, suggesting the validity of the EQ-5D-Y. The differences in the HRQoL across groups by the health behaviour and socio-demographic variables also support the discriminant validity of the EQ-5D-Y in Chinese adolescents.

One strength of the present study is that we analysed the associations between health behaviours and HRQoL with respect of both individual and combined behaviours. Another strength is that the current study used validated questionnaires to assess health behaviours and HRQoL. HRQoL was measured by the EQ-5D-Y, hence, facilitating the comparison of the present study findings with other prior studies using the same HRQoL measure. Additionally, multivariable regression analyses were used to adjust the potential confounding effects of socio-demographic factors of students, thus the association between health behaviours and HRQoL can be considered independent of the effect of students’ gender, grade level and parental education.

Limitations of the study should also be clarified. The observed associations of health behaviours with HRQoL could not be inferred as causality due to the cross-sectional study design. Future research is needed to conduct prospective studies that will help address a prospective relationship between the health behaviour and HRQoL in children and youth. Measurements of health behaviours relied on students’ self-report although questions have been previously validated, hence the findings may be prone to error or bias. The use of objective measures of physical activity (e.g., pedometers) and screen time behaviour (e.g., screen use monitor) would help to make more accurate assessments of these behaviours. However, the use of objective measures may present challenges in financial and human resources’ allocation in large-sample studies. Although the present study analysed five important health behaviours, there may be other health behaviours (e.g., eating fast food) that could impact HRQoL of students. Future research is needed to examine the associations of unmeasured health behaviours in this study with HRQoL. Additionally, the survey was conducted amongst junior high school students in one city in the province, thus the findings in this study are limited to generalise to junior high school students in other areas in China.

The findings in the present study have important implications in public health policy and population health research for improving health behaviours and health amongst children and youth. Public health policymakers, school educators, parents and caregivers of students are encouraged to understand the importance of promoting healthy lifestyle behaviours amongst children and adolescents, and do their best to improve health behaviours of the younger population. Public health strategies should place a priority on those subgroups with poor health behaviours, and target promoting these behaviours together rather than consider them separately. Previous studies suggest that school-health intervention programmes that adopt a comprehensive school health promotion approach incorporating promoting nutrition and diet quality, physical activity and sleep quality, and reducing sedentary behaviours simultaneously may produce more positive effects on health outcomes of children and adolescents (40, 41). According to World Health Organization’s (WHO’s) Health Promoting Schools (HPS) framework (42), school health programmes are needed to implement both at schools and at communities and homes, and target students, their parents and school teachers for the purpose of enhancing HRQoL amongst students. For public health researchers, future research is required to conduct more longitudinal and experimental studies in various socio-economic and cultural contexts to better inform a causal role of the health behaviours for HRQoL of students.

## 5. Conclusion

The present study reveals that sleep time, breakfast eating, physical activity and sedentary behaviour are related to HRQoL amongst junior high school students. Both individual behaviour and their joint behaviours are significantly related to HRQoL amongst students. A dose–response relationship was found in the associations between the health behaviours and HRQoL. The findings suggest that school-based programmes that target promoting these health behaviours amongst adolescents may help to enhance their health and HRQoL. School health programmes that focus on multiple health behaviours simultaneously may be more effective in the improvement of students’ physical and mental health, psycho-social functioning and well-being than targeting single behaviour. Further research using prospective designs, such as cohort studies and school health intervention studies amongst children and adolescents in different cultural, socio-demographic and socio-economic settings, is needed to better understand the relationship between health behaviours and HRQoL amongst children and adolescents.

## Data availability statement

The datasets presented in this article are not readily available because of privacy policies however are available from the corresponding author on reasonable request. Requests to access the datasets should be directed to XYW, wxy196163@163.com.

## Ethics statement

The present study, including the data collection, was approved by Human Research Ethics Boards of the Weifang Medical University. The studies were conducted in accordance with the local legislation and institutional requirements. Written informed consent for participation in this study was provided by the participants’ legal guardians/next of kin, and students themselves.

## Author contributions

XYW conceptualized the study, analyzed data, drafted and revised the manuscript. HQL reviewed the statistical analysis and revised the manuscript. ZHL reviewed the analysis results, drafted and revised the manuscript. TZH conducted data collection, data management and revised the manuscript. YLW, YSY, YR reviewed and revised the manuscript. All authors participated in the critical revisions of the manuscript, and read and approved the final version.

## Footnotes

^1^ https://www.jining.gov.cn/col/col2598/index.html
