# Correction: The individual and combined associations of health behaviours with health-related quality of life amongst junior high school students in China

**DOI:** 10.3389/fpubh.2026.1790305

**Published:** 2026-02-12

**Authors:** Ze Hua Liu, Yi Lin Wang, Yue Shuang Yu, Yan Ren, Tong Zhang, Hong Qing Liu, Xiu Yun Wu

**Affiliations:** 1 School of Public Health, Weifang Medical University, Weifang, Shandong, China; 2 Affiliated Hospital of Jining Medical University, Jining, Shandong, China

**Keywords:** sedentary behaviour, sleep, physical activity, breakfast, health-related quality of life, junior high school students

There was a mistake in Table 1 as published. In the “Variable” column, subheading “Gender,” the published word “Girls” corresponding to frequency number 427 (in the second column) should be corrected as “Boys,” and the word “Boys” corresponding to frequency number 356 (in the second column) should be corrected as “Girls.” The corrected Table 1 appears below.

**Table 1:** Frequency distributions of socio-demographic characteristics, health behaviours of the junior high school students.

**Variable**	**Frequency**	**Percentage, %**
**Using a computer**		
< 2 h/day	645	82.4
≥2 h/day	138	17.6
**Using a cellphone**		
< 2 h/day	520	66.4
≥2 h/day	263	33.6
**MVPA**		
≥2 times a/week	369	47.1
Once a week	179	22.9
Never or once a month	235	30.0
**Eating breakfast**		
Eating everyday	441	56.3
Eating often	226	28.9
Never or almost never eating	116	14.8
**Sleep time**		
≥7 h/day	404	52.7
< 7 h/day	362	47.3
**Number of health behaviours with the unhealthy level response**		
0–1	380	49.6
2	218	28.5
≥3	168	21.9
**Gender**		
Boys	427	54.5
Girls	356	45.5
**Grade level**		
Junior (grade 1 and 2)	478	61.0
Senior (grade 3 and 4)	305	39.0
**Parents' education**		
Junior high school or less	158	20.2
High school	398	50.8
University/College or higher	227	29.0

A correction has been made to the **Results** section, paragraph one. In the published article, there was a mistake in the statement of the percentage of students by gender in the Results section. The percentage was displayed as “Amongst 783 students, 54.5% were girls, 61.0% were in junior grade level.” The correct statement is “Amongst 783 students, 54.5% were boys, 61.0% were in junior grade level.”.

The original version of this article has been updated.

